# Histopathology of an autopsy case of a leadless pacemaker system: a case report

**DOI:** 10.1093/ehjcr/ytae120

**Published:** 2024-03-07

**Authors:** Keigo Misonou, Takahiro Doi, Yusuke Shirai, Daigo Nagahara

**Affiliations:** Department of Cardiology, Cardiovascular Center, Teine Keijinkai Hospital, 1-jo 12-chome, Maeda, Teine-ku, Sapporo, Japan; Department of Cardiology, Cardiovascular Center, Teine Keijinkai Hospital, 1-jo 12-chome, Maeda, Teine-ku, Sapporo, Japan; Department of Cancer Pathology, Faculty of Medicine, Hokkaido University, Sapporo, Japan; Department of Cardiology, Cardiovascular Center, Teine Keijinkai Hospital, 1-jo 12-chome, Maeda, Teine-ku, Sapporo, Japan

A 60-year-old man presented to a local hospital with chest pain. A computed tomography (CT) scan revealed sub-endocardial left ventricular contrast defects in the lateral wall region and pericardial effusion (*Figure 1A*). Transthoracic echocardiography performed after transport to our hospital revealed abnormal wall motion of the lateral region of the left ventricle and pericardial effusion at the posterior region of the left ventricle.

**Figure 1:**
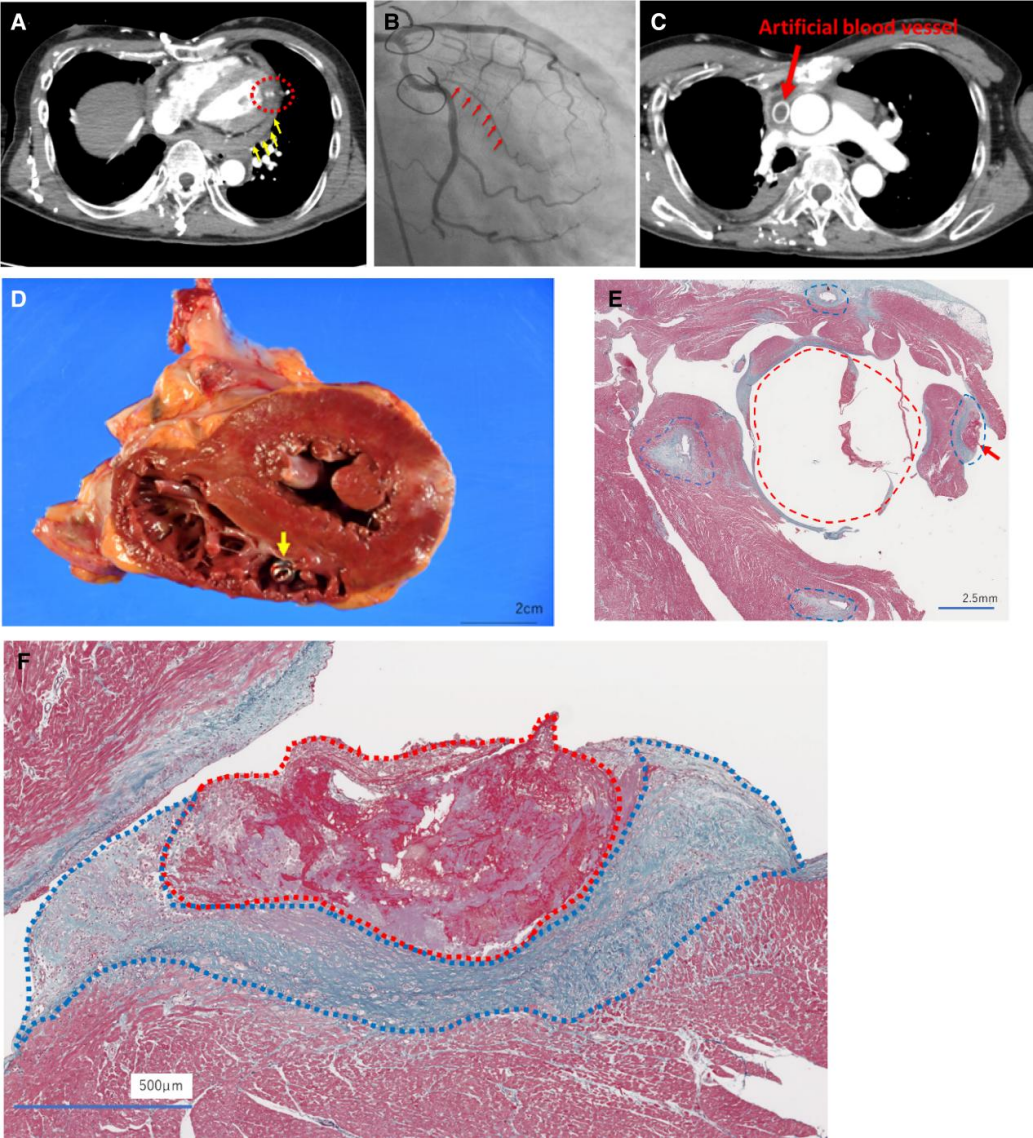
(*A*) Poor contrast image in the left ventricular lateral wall myocardium from the endocardium to the epicardium, leakage of contrast medium outside the heart, and pericardial effusion on contrast-enhanced computed tomography at the previous hospital. Inside of the red dotted line: site of myocardial infarction. Yellow arrow: pericardial effusion. (*B*) Thrombolysis in myocardial infarction (TIMI) 2 (red arrow) in Seg12-2 of the left coronary artery circumflex branch on emergency coronary angiography. (*C*) Findings suggestive of occlusion of the superior vena cava and the replaced prosthesis on contrast-enhanced computed tomography at the previous hospital. (*D*) The leadless pacemaker system implanted in the right ventricular myocardium on autopsy tissue. Yellow arrow: the main body of the leadless pacemaker. (*E*) Surrounding tissue of the implanted leadless pacemaker system between the papillary muscles at autopsy (Elastica–Masson stain). Red dotted line area: the hole where the leadless pacemaker system was implanted. Blue dotted line area: the site of the hole where the hook of the leadless pacemaker system was hooked into the myocardium. (*F*) Higher magnification of the area indicated by the red arrow in *E*. Thrombus formation with fibrous thickening of the endothelium and infiltrating inflammatory cells were observed around the hook of the leadless pacemaker system. There is an increase in collagen fibres at the thrombus margin, but the thrombus is stained red, and a small number of macrophages remain in the centre, suggesting that it is a relatively new thrombus (Elastica–Masson stain). Red dotted line area: the thrombus around the hook of the leadless pacemaker system. Blue dotted line area: the infiltrating inflammatory cells around the hook of the leadless pacemaker system.

Coronary angiography showed 99% stenosis in the left circumflex artery (*Figure 1B*). Considering the small size of the myocardial infarction and concomitant cardiac oozing rupture, conservative management was chosen. However, administration of amiodarone and beta-blockers for the treatment of refractory supraventricular and ventricular tachycardia resulted in sudden cardiac arrest due to sinus arrest on the 46th day. He became pacemaker dependent, and a leadless pacemaker system (LPS) was implanted on the 53rd day because a CT scan showed occlusion of an artificial vessel in the superior vena cava, which had been replaced 30 years earlier for treatment of a thymoma^1,2^ (*Figure 1C*).

However, he died of pump failure from diastolic dysfunction of both ventricles due to marked epicardial fibrosis 1 month after implantation of the pacemaker.

Autopsy showed that the LPS was implanted in the right ventricular myocardium (*Figure 1D*). Elastica–Masson staining (*Figure 1E* and *F*) revealed thrombus formation around the site of implantation. Endocardial fibrosis and inflammatory cell infiltration were also observed around the thrombus.

The removability of new LPSs after long-term implantation is a major problem that has yet to be resolved.^3^ At autopsy in this case, the LPS was covered with fibrotic tissue on the surface of the implantation site even only 4 weeks after implantation, raising questions about the removability of the device even after a short period. The indication for device retrieval should be carefully considered in cases with a long period after implantation.

## Data Availability

The data will be shared on reasonable request to the corresponding author.
